# Notch activation stimulates migration of breast cancer cells and promotes tumor growth

**DOI:** 10.1186/bcr3447

**Published:** 2013-07-04

**Authors:** Victoria Bolós, Emilia Mira, Beatriz Martínez-Poveda, Guillermo Luxán, Marta Cañamero, Carlos Martínez-A, Santos Mañes, José Luis de la Pompa

**Affiliations:** 1 Program of Cardiovascular Developmental Biology, Department of Cardiovascular Development and Repair, Centro Nacional de Investigaciones Cardiovasculares (CNIC), Melchor Fernández Almagro 3, E-28029 Madrid, Spain; 2 Department of Immunology and Oncology, Centro Nacional de Biotecnología/CSIC, Darwin 3, Campus de Cantoblanco, E-28049 Madrid, Spain; 3 Comparative Pathology Unit, Biotechnology Program, Centro Nacional de Investigaciones Oncológicas, E-28029 Madrid, Spain

**Keywords:** Mammary tumor, MCF-7, HT-29, MDA-MB-231, NOTCH, E-CADHERIN, EMT, migration, growth

## Abstract

**Introduction:**

Dysregulated NOTCH receptor activity has been implicated in breast cancer but the mechanisms by which NOTCH contributes to transformation are not yet clear, as it has context-dependent effects on the properties of transformed cells.

**Methods:**

We have used various *in vitro *and *in vivo *carcinogenic models to analyze the impact of Notch signaling in the onset and progression of breast tumors.

**Results:**

We found that ectopic expression of the Notch1 intracellular domain (N1ICD) in MCF-7 breast adenocarcinoma cell line caused reduction and delocalization of E-CADHERIN levels and increased migratory and invasive abilities. Notch inhibition in the invasive breast cancer cell line MDA-MB-231 resulted in increased E-CADHERIN expression and a parallel reduction in their invasive capacity. The growth of subcutaneous xenografts produced with MCF-7 cells was boosted after N1ICD induction, in a cell autonomous manner. *In vivo *Notch1 activation in the mammary gland using the *MMTV-Cre *driver caused the formation of papillary tumors that showed increased *Hes1 *and *Hey1 *expression and delocalized E-cadherin staining.

**Conclusions:**

These results confirm NOTCH1 as a signal triggering epithelial-mesenchymal transition in epithelial cancer cells, which may have implications in tumor dissemination, metastasis and proliferation *in vivo*. The identification of specific factors interacting with NOTCH signaling could thus be relevant to fully understanding the role of NOTCH in breast neoplasia.

## Introduction

Notch is a fundamental signaling pathway that regulates embryonic cell fate specification, proliferation and patterning [1,2]. In addition to its central role in development, Notch signaling is deregulated in a number of cancers [3]. *Notch1 *mutations lead to oncogene expression in certain T cell acute lymphoblastic leukemias [4] and a subset of breast carcinomas [5]; deregulated Notch activity might also affect cell transformation [6], regulation of the cell cycle [7], progenitor/stem cell maintenance [3] and the outcome of breast cancer [8].

The mammalian Notch proteins (Notch1 to 4) are membrane-bound type I receptors with a large extracellular domain involved in ligand binding, and a cytoplasmic domain responsible for signal transduction. The Notch ligands Delta-like 1, 3 and 4 and Jagged 1 and 2 are also membrane-bound. Ligand-receptor interactions between neighboring cells trigger Notch signaling, which leads to a sequence of proteolytic cleavage events in the receptor. The last of these is mediated by γ-secretase activity, generating the Notch intracellular domain (NICD), which translocates to the nucleus and binds the CSL transcription factor. The NICD/CSL complex induces expression of target genes, including those of the hairy/enhancer of split (*Hes*) family [1,2], the cell cycle regulator *p21 *[9] and *cyclin D1 *[7].

Many studies focus on the role of Notch1 in mammary tumorigenesis. Hyperactivated Notch1 signaling was first implicated in mammary tumorigenesis in studies of the MMTV model, which showed that N1ICD expression in *MMTV-Neu *mammary tumors is due to an MMTV insertion in the *Notch1 *locus [10]. Other reports indicated that transgenic activation of N1ICD in mammary glands leads to development of lactation-dependent tumors that regress at weaning [11,12]. These findings link aberrant Notch activation in the murine mammary gland to adenocarcinoma. Experimental evidence shows that altered Notch1 signaling leads to direct transcriptional regulation of *c-myc*, which is crucial in *MMTV-N1ICD*-induced murine mammary tumorigenesis [13]. NOTCH1 is also involved in human mammary tumorigenesis as a downstream effector of oncogenic Ras [14].

Here we used various *in vitro *and *in vivo *models to analyze the impact of Notch signaling in breast tumor onset and progression. We find that stable or inducible N1ICD expression in the poorly invasive MCF-7 breast adenocarcinoma cell line causes a reduction and delocalization of E-CADHERIN levels, suggesting a disassembly of adherens junctions that correlates with enhanced cell migratory and invasive abilities. These properties may be extended to other epithelial tumor cell lines as we have made similar observations in the colon cancer cell line HT-29 stably expressing N1ICD. To the contrary, Notch inhibition in the highly invasive cell line MDA-MB-231 resulted in increased E-CADHERIN expression and a parallel reduction in their invasive capacity. Notch1 activation in the mouse mammary gland using the *MMTV-Cre *driver caused the formation of papillary tumors that showed increased Hes1 and *Hey1 *and delocalized E-cadherin expression. We also found that the growth of subcutaneous xenografts produced with MCF-7 cells was boosted after N1ICD induction, in a cell autonomous manner. These results confirm Notch1 as an epithelial-to-mesenchymal transition (EMT) inducer in breast cancer cells, which may have implications in tumor dissemination and metastasis.

## Methods

### Cell lines

The human breast cancer cell lines MCF-7 (ATCC® HTB-22™) and MDA-MB-231 (ATCC® HTB-26™), and the human colorectal adenocarcinoma cell line HT-29 (ATCC® HTB-38™) were used. For culture conditions see Additional file 1, Supplementary Materials and methods.

### Transfection of MCF-7 and HT-29 cells

A cDNA fragment encoding the active version of mouse Notch1 (N1ICDΔ^OP^) was used [15]. The Tet-Off system was employed to obtain transfectants of MCF-7 with inducible N1ICD expression. In this system, gene expression is turned on when doxycycline (DOXY; a tetracycline derivative) is removed from the culture medium. For details see Additional file 1, Supplementary Materials and methods.

### Western blot analysis

For details see Additional file 1, Supplementary Materials and methods.

### Semi-quantitative RT-PCR and real-time quantitative PCR

Total RNA was extracted with Trizol reagent (Life Technologies, NY, USA) and cDNA was synthesized with SuperScript III First Strand kit (Life Technologies, NY, USA). *N-Cadherin *primers were 5´-CACCCAACATGTTTACAATCAACAATGAGAC-3 (forward) and 5´-CTGCAGCAACAGTAAGGACAAACATCCTATT-3 (reverse) [16]. Commercial β-actin primers were used (Stratagene, La Jolla, CA, USA). Quantitative PCR was performed with Power SYBR Green Master Mix (Applied Biosystems, NY, USA, 4367659) and commercial primers for *HEY1, HES1, cMYC, NOTCH1, NOTCH4, SNAI1, ECAD, VIMENTIN *and *HPRT1 *(Sigma, St. Louis, MO, USA) were used.

### Promoter activity assays

*Hes1-Luc *promoter activity [17] was measured in MCF-7 cells expressing N1ICD in a constitutive or inducible manner. The activity of the artificial promoter 10XCBF1 [18] was measured after transient transfection of MDA-MB-231 cells. Briefly, cells were co-transfected with the plasmid containing the promoter *10XCBF1-Luc *and pcDNA3-CBF1-VP16 or pcDNA3-DN-CBF1/RBPJK. The plasmid pTK-RL (Promega, Madison, WI, USA)

was also included as a control of transfection efficiency. When indicated, cells were treated for the indicated period of time with DOXY 2 μg/ml or with the γ-Secretase Inhibitors DAPT (N-(N-(3,5-Difluorophenacetyl)-L-alanyl)-S-phenylglycine t-butyl ester, 10 to 50 μM; 565770, Calbiochem, Millipore, MA, USA) and RO4929097 ((2,2-dimethyl-N-(S)-6-oxo-6,7-dihydro-5H-dibenzo(b,d)azepin-7-yl)-N'-(2,2,3,3,3-pentafluoro-propyl)-malonamide)), 10 to 20 μM; S1575, Selleckchem, Houston, TX, USA) for 48 h. After transfection cells were lysed with passive lysis buffer (Promega, Madison, WI, USA) and firefly and renilla luciferase were measured with the "Dual-luciferase reporter assay" (Promega, Madison, WI, USA). The activity in MCF-7 clones or in MDA-MB-231 treated cells was referred to the activity in control cells or cells transfected with the empty vector (pcDNA3).

### Immunofluorescence and immunohistochemistry

For details see Additional file 1, Supplementary Materials and methods.

### *In situ *hybridization

*In situ *hybridization was performed as described in [19]. Details of probes will be provided on request.

### *In vitro *cell chemotaxis

Cell migration was performed in Transwell (Corning, Tewksbury, MA, USA) with 8 μm pore filters coated with 20 μg/ml collagen type IV (Sigma, St. Louis, MO, USA). Cells were pretreated for the time indicated with DOXY (MCF-7) or DAPT/RO4929097/DMSO (MDA-MB-231), trypsinized and added to the upper chamber in basal medium with 0.5% BSA and the additives. The lower chamber was replenished with basal medium with BSA and the chemoattractant (IGF-1, 50 ng/ml, R&D Systems, Minneapolis, MN, USA or SDF1α, PeProTech (New Jersey, USA). After 18 h incubation, the upper chamber was emptied and cells remaining are removed. Cells in the filter are fixed with PFA and then stained with violet crystal (Sigma-Aldrich). Cell counts were obtained by counting two (MDA-MB-231) or four (MCF-7) grids using a microscope fitted with a grid eyepiece at a total magnification of 100X.

### Flow cytometry

For details see Additional file 1, Supplementary Materials and methods.

### *In vivo *experiments with mice

All animal procedures were approved by the Institutional Committee for the Care and Use of Laboratory Animals of the Centro Nacional de Investigaciones Cardiovasculares (CNIC, Madrid, Spain) and Centro Nacional de Biotecnología (CNB-CSIC, Madrid, Spain). Animal procedures conformed to EU Directive 2010/63EU and Recommendation 2007/526/EC, regarding the protection of animals used for experimental and other scientific purposes, enforced in Spanish law under Real Decreto 1201/2005.

### Tumorigenic assays

MCF-7/TetOff and B12, M5 and M20 derivatives' clones, growing in culture without DOXY for 25 days, were inoculated s.c in both flanks (1.5 or 2.4 × 10^6 ^cells) in BALBc/SCID mice treated with 17α-ethylenestradiol 1 μg/ml (Sigma) provided in the drinking water from one week before cells were injected. Tumor size was monitored weekly and tumor volume estimated with a caliper by measuring the width (a) and the length (b) and applying the formula (a^2 ^× b)/2. Once finished with the period of treatment, mice were sacrificed and tumors were extracted for further analysis. MCF-7/TetOff and B12 were transduced with recombinant retrovirus to express luciferase activity. Plasmid pRV-luc-IRES-CopGreen was used to obtain the retroviral supernatants (Genetrix S.L., Madrid, Spain) and transduced cells were sorted according to the associated green fluorescence. BALBc/SCID mice (Harlan Laboratories, Indianapolis, IN, USA) were injected in the two inguinal mammary glands with 2.5 × 10^6 ^cells and mice were treated as above. After injection, half of the mice were treated also with DOXY 2 mg/ml provided in the drinking water. Tumoral growth rate was analyzed by bioluminescence at different weeks after cell inoculation. Briefly, mice were injected with luciferin with the general anesthetic and luciferase activity expressed by cells was detected with a CCD camera placed in a dark box (Hamamatsu Photonics, Shizuoka, Japan). Images were processed with the software provided and luminescence units were represented. Tumor size was estimated as above and once finished with the period of treatment, tumors were excised, weighted and preserved adequately to make further analysis.

### Transgenic N1ICD expression in the mammary gland

The transgenic lines *MMTV-Cre *[20] and *Rosa26N1ICD *[21] were bred to generate *MMTV-Cre/+; Rosa26N1ICD/+ *double transgenic mice. For primers and conditions of mouse genotyping see [20,21]. Mice were subjected to several rounds (a median of four) of pregnancy and lactation, and when a breast tumor arose, mice were euthanized and the breast tumor excised and processed for further analysis. Tumor samples were fixed with 10% buffered formalin (Sigma-Aldrich) for 48 h and afterward were paraffin-embedded. Staining of Hes1, ERα, p63, E-cadherin and Ki67 was performed in 5 μm sections of paraffin samples following standard techniques. For details see Additional file 1, Supplementary Materials and methods.

## Results

### N1ICD expression enhances the invasive capacity of the breast cancer cell line MCF-7

**Figure 1:**
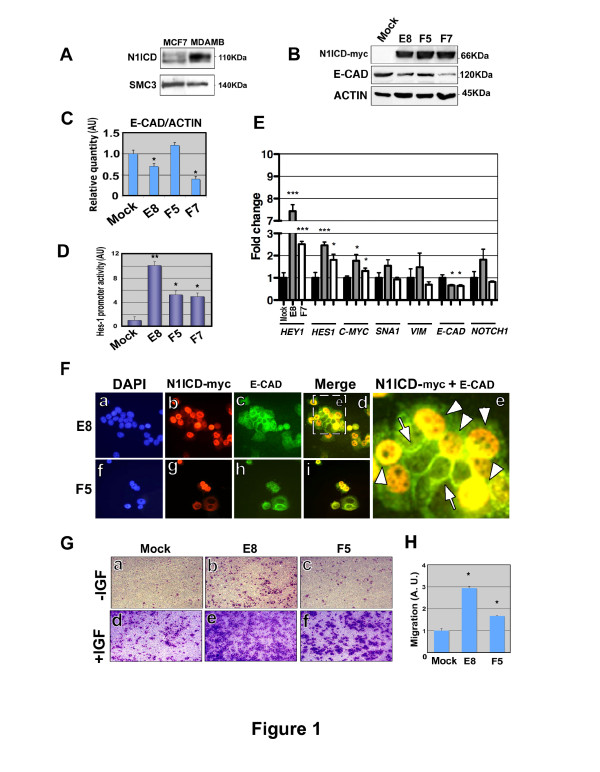
**Stable N1ICD expression induces migration of MCF-7 cells**. (**A**) Western blot showing expression of endogenous N1ICD in MCF-7 and MDABM231 cells. SMC3 (Structural Maintenance of Chromomosomes-3 protein) was used as a loading control. (**B**) Expression of N1ICD and E-CADHERIN in the stable MCF-7 clones E8, F5 and F7 besides with mock MCF-7 cells; β-ACTIN was used as a loading control. (**C**) The Western blot in (B) was quantified by densitometry and the ratio of E-CADHERIN/β-ACTIN calculated. (**D**) NOTCH activity was determined with a *Hes-1 *promoter fragment. The value in the clones is represented relative to the value in mock cells. Data are mean ± SEM of quadruplicates in three independent experiments (*P <0.05 determined by Student's *t *test). (**E**) Expression of *HEY1, HES1, C-MYC, SNAI1, VIMENTIN, E-CADHERIN *and *NOTCH1 *in the stable MCF-7 clones E8 and F7, measured by qPCR. Data are mean ± SEM of triplicates in two independents experiments (*P <0.05, ***P <0.001 determined by Student's *t-*test). (**F**) Staining of N1ICD (red), E-CADHERIN (green) and nuclei (DAPI, blue) in E8 and F5 MCF-7-N1ICD clones. Arrows indicate the intercellular staining of E-CADHERIN and arrowheads its intracellular accumulation. (**G, H**) Effect of ectopic N1ICD expression on the chemotaxis of E8 and F5 MCF-7 cell clones. (G) Representative images of transmigrated cells in basal (-IGF-1) and after IGF-1 addition (+IGF). (H) Quantification of the transwell assays. The migration index was calculated for each condition, and then referred to that of mock (considered as 1). Data are mean ± SEM of triplicates in three independent experiments (*P <0.05 determined by Student's *t *test). DAPI, 4,6-Diamidino-2-phenylindol; MCF-7, Michigan Cancer Foundation-7 breast cancer cell line; N1ICD, Notch one intracellular domain

To gain an insight into the role of Notch in breast cancer we used the breast cancer cell line MCF-7 that has several features of differentiated mammary epithelium [22]. These cells show low levels of N1ICD expression by Western blot when compared with the metastatic breast cancer cell line MDA-MB-231 (Figure 1A). This observation fits with the idea that high NOTCH signaling is associated with the expression of basal breast cancer markers [23].

We generated MCF-7 clones stably expressing a myc-tagged N1ICD version (Figure 1B). MCF-7 cells have a typical cobblestone phenotype (not shown) and express the epithelial cell marker E-CADHERIN (Figure 1B, C). N1ICD expression caused a reduction in total E-CADHERIN levels in MCF-7 clones E8 and F7 but not in clone F5 (Figure 1B, C). The levels of Notch activity in MCF-7/N1ICD cells measured by a luciferase reporter assay using a fragment of the mouse *Hes1 *promoter [17], revealed an evident activation of the Notch pathway in comparison with control, mock-transfected MCF-7 cells (Figure 1D). qPCR analysis revealed a marked up-regulation of the NOTCH target genes *HEY1, HES1 *and *C-MYC *while the epithelial marker *E-CADHERIN *was down-regulated and *SNAIL1 *and *VIMENTIN *were not significantly changed (Figure 1E). We also examined *NOTCH1 *and *NOTCH4 *expression because of their role in mouse breast cancer malignancy [24] and their overexpression in triple-negative breast cancer subtypes [25]. *NOTCH4 *expression was almost undetectable in MCF-7 cells (Additional file 2, Figure S1A) while *NOTCH1 *expression was unaffected (Additional file 2, Figure S1B and not shown), as previously reported [26]. Semi-quantitative RT-PCR analysis of various MCF-7-N1ICD expressing clones revealed no variation in *JAG1 *and *TWIST1 *expression (Additional file 2, Figure S1B). The lack of response of *TWIST1 *to N1ICD expression is in agreement with previous findings showing that during developmental EMT, *Twist1 *is induced by Bmp2 [27] but does not respond to Notch [28]. Immunofluorescence analysis confirmed that forced N1ICD expression caused a reduction in membranous E-CADHERIN staining (Figure 1Fa-i). Cells with strong nuclear N1ICD staining showed mostly nuclear E-CADHERIN expression, suggesting a disassembly of adherens junctions [29], which contrasted with its accumulation in the membrane at the cell-cell contacts of MCF-7 cells that did not express N1ICD (Figure 1Fe). Concomitant to the reduction in E-CADHERIN levels, N1ICD expression endowed MCF-7 cells with increased chemotactic ability towards IGF-1 (Figure 1G, H), a chemo-attractant for this cell line [30]; some N1ICD-expressing MCF-7 clones showed an increased migratory capacity, even in basal medium (Figure 1Gb, c). To test if the reduction of *E-CADHERIN *upon N1ICD expression could be extended to other epithelial tumor cell lines, we transfected N1ICD into the HT-29 colon adenocarcinoma cell line and generated stable clones. We chose HT-29 cells because, similarly to the mammary gland, they derive from a tissue in which NOTCH has an oncogenic role [31,32]. Additional file 3, Figure S2A, B shows that HT-29 cells stably expressing N1ICD down-regulate E-CADHERIN expression.

### Inducible N1ICD expression in MCF-7 cells leads to *E-cadherin *down-regulation and increased migratory capacity

**Figure 2:**
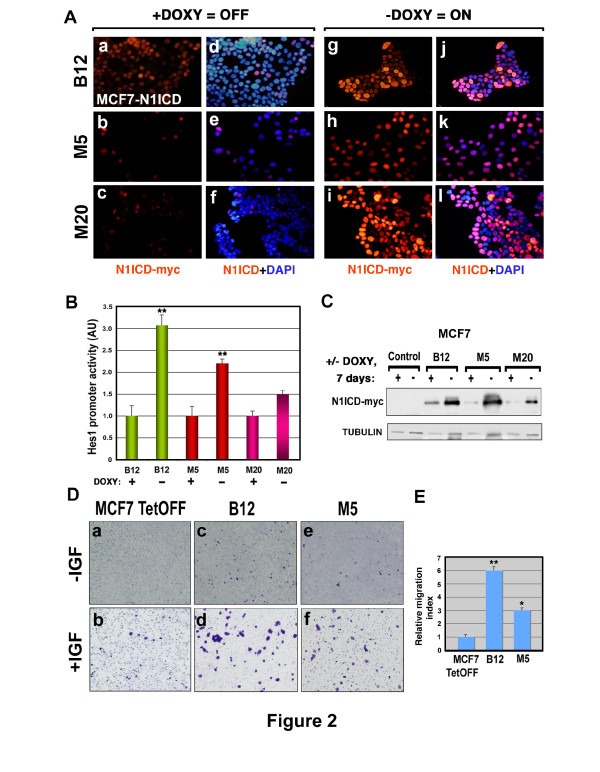
**Inducible N1ICD overexpression in MCF-7 cells promotes a migratory behavior**. (**Aa-f**) General view of the MCF-7 Tet-Off clones (B12, M5 and M20) cultured in the presence of DOXY. a-c, anti-myc staining reveals leaky myc-N1ICD expression; d-f, anti-myc and DAPI staining overlapping. (**Ag-l**) General view of these clones cultured in the absence of DOXY. g-i, anti-myc staining reveals N1ICD induction in these cells; j-l, anti-myc and DAPI staining. (**B**) Quantification of *Hes1 *promoter activity in MCF-7 uninduced and induced clones. Data are mean ± SEM of quadruplicates in three independent experiments (**P <0.005 determined by Student's *t-*test). (**C**) Western blot analysis of myc-N1ICD expression in MCF-7 control cells and MCF-7 clones after seven days of culture with or without DOXY. (**D**) Representative images of transmigrated cells in basal (-IGFI) and IGF-I supplemented (+IGF) cells. (a, b) control, (c, d) N1ICD-induced B12 and (e, f) M5 MCF-7 cells. (**E**) The relative migration index for each cell type is represented. Data are mean ± SEM of duplicates in three independent experiments (*P <0.05, **P <0.005 determined by Student's *t *test). DAPI, 4,6-Diamidino-2-phenylindol; DOXY, doxycycline; MCF-7, Michigan Cancer Foundation-7 breast cancer cell line; N1ICD, Notch one intracellular domain

To study more precisely the effect of Notch expression in MCF-7 cells, we generated N1ICD-inducible clones using the Tet^OFF ^system, so that gene expression was induced when the antibiotic doxycycline (DOXY) was removed from the culture medium. Figure 2A shows N1ICD-myc staining of three inducible clones (B12, M5 and M20) cultured in the presence (OFF condition) or absence (ON condition) of DOXY. There was some leaky N1ICD expression in the OFF condition in these three clones (especially for B12; Figure 2Aa-f), but 48 h after DOXY withdrawal there was a clear induction of N1ICD expression (Figure 2Ag-l), although not in 100% of the cells. Examination after seven days of induction also revealed a non-homogenous N1ICD expression in these MCF-7 clones (data not shown).

The effect of DOXY retrieval in N1ICD induction was measured by luciferase assay upon transfection of a *Hes1 *reporter. There was clear reporter activation after N1ICD induction in the different clones studied, especially in clone B12 at 48 h (Figure 2B). This enhanced N1ICD-induced transcriptional activity correlated with the increase of N1ICD expression in the different clones upon doxycycline withdrawal (Figure 2C). Concomitantly to N1ICD induction, there was an increase in the migratory capacity of these cells (Figure 2D, E).

**Figure 3:**
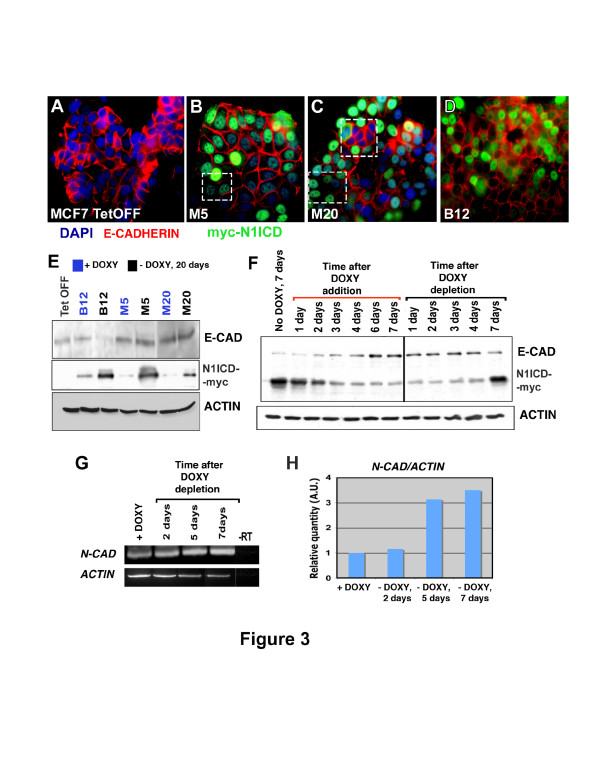
**E-CADHERIN and N-CADHERIN levels are modified in MCF-7 cells by overexpression of N1ICD**. (**A**) Immunofluorescence staining of E-CADHERIN expression (red) in MCF-7 Tet-Off cells. (**B-D**) E-CADHERIN staining is lost in some cells where N1ICD (green) is induced. Nuclei are counterstained with DAPI (blue). (**E**) Western blot analysis of E-CADHERIN in MCF-7 mock and MCF-7 inducible clones in the presence (blue) or absence (black) of doxyxycline. (**F**) Time-course analysis of E-CADHERIN expression by Western blot after DOXY addition and depletion in clone B12. (**G, H**) *N-CADHERIN *transcription measured by RT-PCR in clone B12 after DOXY depletion. DAPI, 4,6-Diamidino-2-phenylindol; DOXY, doxyxycline; MCF-7, Michigan Cancer Foundation-7 breast cancer cell line; N1ICD, Notch one intracellular domain

To investigate the possible correlation between the enhancement in the migratory capacity of MCF-7-Tet^OFF^-N1ICD clones and the reduction of E-CADHERIN expression in these cells, we performed an immunofluorescence analysis of clones M5, M20 and B12 after 20 days of culture in the absence of DOXY. Induction of N1ICD expression coincided with a reduction of membranous E-CADHERIN staining in these clones (Figure 3A-D). Western blot analysis after 20 days of induction revealed a marked reduction in E-CADHERIN expression in clone B12 that was not so apparent in clones M5 and M20 (Figure 3E). We then carried out a time-course of N1ICD repression/induction in clone B12 and its effect on E-CADHERIN expression. As Figure 3F shows, there was an inverse correlation between N1ICD and E-CADHERIN expression. MCF-7-Tet^OFF^-N1ICD cells of clone B12 cultured seven days in the absence of DOXY showed strong N1ICD expression (Figure 3F). After one day in culture in the presence of DOXY, N1ICD expression was progressively reduced and in parallel, E-CADHERIN expression was increased throughout seven days of culture (Figure 3F). To the contrary, when cells from clone B12 were grown in the absence of DOXY, N1ICD expression was increased and E-CADHERIN expression was reduced, and this effect was clear after seven days of culture without DOXY (Figure 3F).

These data indicated that N1ICD expression in MCF-7 cells, either in an inducible or stable manner, leads to a reduction in E-CADHERIN levels, suggesting that these cells began to lose their epithelial phenotype. During EMT there is a progressive cadherin switch, such that E-cadherin expression is reduced and N-cadherin expression is increased [33]. Figure 3G shows a semi-quantitative RT-PCR analysis of *N-CADHERIN *expression in clone B12. Upon DOXY withdrawal there was a progressive increase in *N-CADHERIN *expression that was at a maximum after seven days of culture (Figure 3G, H), suggesting that B12 cells acquired a mesenchymal phenotype. Moreover, we found that N1ICD induction enhanced VIMENTIN expression although it did not change *Twist1 *mRNA levels (Additional file 4, Figure S3).

### NOTCH inhibition reduces the migratory ability of MDA-MB-231 cells

**Figure 4:**
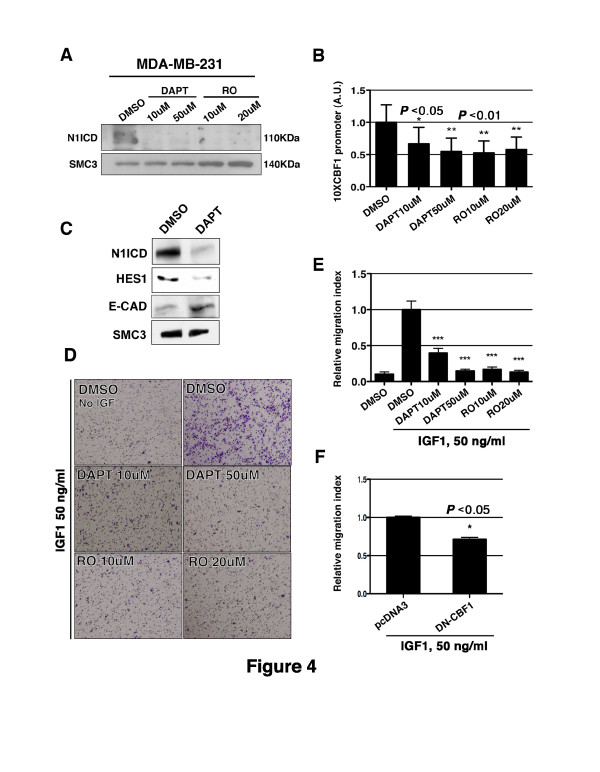
**NOTCH signaling inhibition decreases the migratory behavior of MDA-MB-231 cells**. (**A**) Western blot showing Notch one intracellular domain (N1ICD) expression in MDA-MB-231 cells after 48 h of treatment with DAPT or RO4929097 (RO). Equal amounts of protein loading were validated with an anti-SMC3 antibody. (**B**) Notch activity measured with the 10 × CBF1 reporter. The value for DAPT and RO4929097 treated cells is relative to the activity in cells treated with DMSO. Data are mean ± SEM of quadruplicates in three independent experiments (*P <0.05, **P <0.005 determined by Student's *t-*test). (**C**) Representative images of migration towards IGF-1 50 ng/ml of MDA-MB-231 cells with DAPT or RO4929097 or the vehicle DMSO. (**D**) Migration index obtained in both conditions. Data are mean ± SEM of duplicates in three independent experiments (***P <0.001 determined by Student's *t-*test). (**E**) Migration index quantification of MDA-MB-231 cells transfected with an empty vector (pCDNA3) or a dominant negative version of CBF1 (DN-CBF1) towards IGF-1 (50 ng/ml). Data are mean ± SEM of duplicates in three independent experiments (*P <0.05 determined by Student's *t-*test). CBF1, CSL, Suppressor of Hairless; DAPT, N-(N-(3,5-difluorophenacetyl)-l-alanyl)-S-phenylglycine t-butyl ester; DMSO, dimethyl sulfoxide; MCF-7, Michigan Cancer Foundation-7 breast cancer cell line; MDA-MB-231, Breast cancer cell line derived from metastatic site (pleural effusion); N1ICD, Notch one intracellular domain

To gain further insights on the role of NOTCH in breast tumor progression and the potential use of NOTCH inhibitors as therapeutic agents against breast cancer, we inhibited NOTCH activity in the adenocarcinoma cell line MDAMB-231 [34], which is more tumorigenic and invasive than the MCF-7 cell line. MDA-MB-231 cells endogenously expressed N1ICD (Figures 1A and 4A) and E-CADHERIN levels in MDA-MB-231 were reduced compared to those of MCF-7 cells (Additional file 5, Figure S4). We used the γ-secretase inhibitors DAPT [35] and RO4929097 [36], to prevent the generation of NICD and thus inhibit Notch activity. After 48 h we could readily detect a drastic reduction in N1ICD levels in DAPT- and RO-treated cells (Figure 4A). This effect could also be measured by the reduction in the activity of a CBF1 reporter (Figure 4B). NOTCH inhibition also resulted in an up-regulation of E-CADHERIN paralleled by a reduction in HES1 expression (Figure 4C). The migratory response of MDA-MB-231 cells towards IGF-1 was reduced around 80% when cells were cultured with DAPT or RO (Figure 4D, E). MDA-MB-231 cells were also transfected with a dominant-negative version of CBF1 (DN-CBF1); we observed a 25% reduction in the migratory capacity of DN-CBF1-expressing MDA-MB-231 cells compared to controls (Figure 4F). The weaker inhibition of migration in comparison with the DAPT or RO treatments was likely due to the fact that MDA-MB-231 cells were transiently transfected and only a subpopulation expressed the DN-CBF1 construct (data not shown).

### Inducible Notch1 activation in MCF-7 cells stimulates tumor growth *in vivo*

**Figure 5:**
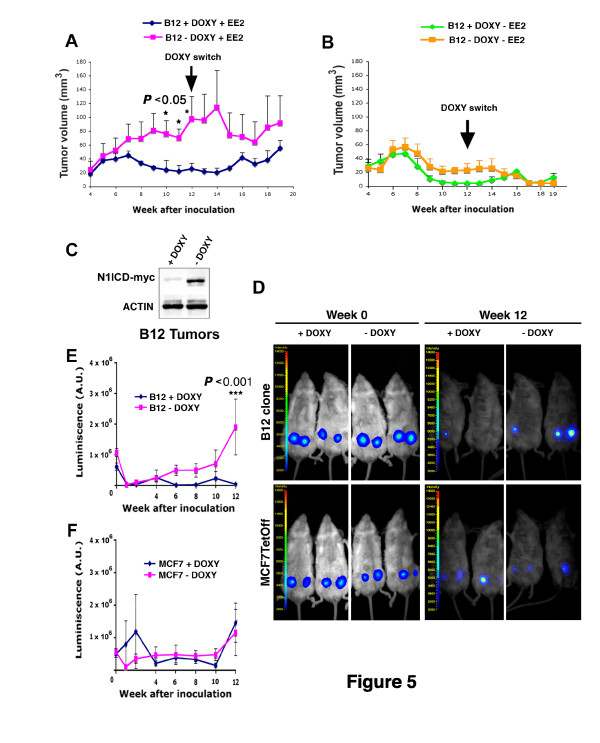
**N1ICD induction promotes a tumorigenic behavior**. (**A**) Growth of xenografts derived from MCF-7 B12 clone with induced N1ICD (-Doxy) or without N1ICD (+Doxy) expression. Mice also had 17α-ethynyl estradiol (+EE2) in the drinking water. The arrow indicates the 12^th ^week when DOXY treatment was switched between both groups of mice. (**B**) Growth of N1ICD-induced B12 cells in the absence of estrogen (+EE2) administration. The arrow indicates the week 12^th ^when DOXY treatment was switched. Data are mean ± SEM of *n *= 8 (four mice and two tumors per mice; *P <0.05 determined by two-way ANOVA with Bonferroni post-test). (**C**) Western blot showing expression of myc-tagged N1ICD in B12 tumors at 12^th ^week of cell inoculation. (**D**) MCF-7 Tet-Off or the MCF-7 B12 clone engineered to express luciferase injected in the mammary gland of BALBc/SCID mice were monitored by bioluminescence emission at the time of cell inoculation and after 12 weeks as indicated. Selected images of two independents experiments, four animals per group. (**E**) Growth dynamics measured by luminescence of MCF-7 B12 clone with (-DOXY) or without (+DOXY) N1ICD expression. (**F**) Growth of control MCF-7 clones in the same conditions. Data are mean ± SEM of *n *= 8 (four mice and two tumors per mice; ***P <0.001 determined by two-way ANOVA with Bonferroni post-test), representative of two independents experiments. DOXY, doxyxycline; MCF-7, Michigan Cancer Foundation-7 breast cancer cell line; N1ICD, Notch one intracellular domain

To examine the tumorigenic ability of MCF-7-N1ICD inducible clones we focused on the B12 clone as it showed a clear phenotypic change as a consequence of N1ICD induced overexpression. Growth of MCF-7 xenografts is estrogen-dependent [37], so we injected MCF-7-Tet-Off-N1ICD B12 cells and added 17α-ethynyl estradiol to the drinking water. When small size tumors were evident in all injection points, mice were divided into two groups. In the first group N1ICD expression was "turned on" (no DOXY) and in the second one, N1ICD expression was turned "Off" by addition of DOXY to the drinking water. As Figure 5A shows, the tumors in which N1ICD expression was turned off did not grow significantly, while tumors in which N1ICD expression was maintained for 12 weeks continued growing and were significantly larger than those generated by control MCF-7 (data not shown) or by un-induced cells (Figure 5A). After 12 weeks, treatments were switched between both groups of mice (Figure 5A, arrow), and tumors were monitored for seven weeks more. The result was a reduction in the differences between both groups, leading to similar tumor size (Figure 5A). Thus, tumor growth was clearly dependent on turning "On" or "Off" N1ICD expression.

The growth curves shown in Figure 5A, B suggest also that estrogens may be a limiting factor in Notch-mediated tumor formation and growth. Western blot analysis of xenografts generated after 12 weeks of N1ICD induction revealed strong N1ICD expression (Figure 5C).

Next, we analyzed growth of orthotopic tumors formed by the clone B12 transduced with a luciferase-expressing retrovirus to monitor tumor evolution by chemoluminiscence. After cell injection, mice were separated in two groups, receiving (N1ICD off) or not (N1ICD on) DOXY in the drinking water. In agreement with our previous results (Figure 5A), clone B12 with induced N1ICD expression gave rise to tumors significantly larger than those generated when N1ICD was not expressed (Figure 5D, E). DOXY treatment did not affect the growth of tumors formed by control MCF-7 cells (Figure 5D, F). These results suggested that N1ICD induction might be directly responsible for MCF-7 tumor formation.

### N1ICD expression in the mammary gland leads to tumor formation and reduction in estrogen receptor and E-cadherin expression

**Figure 6:**
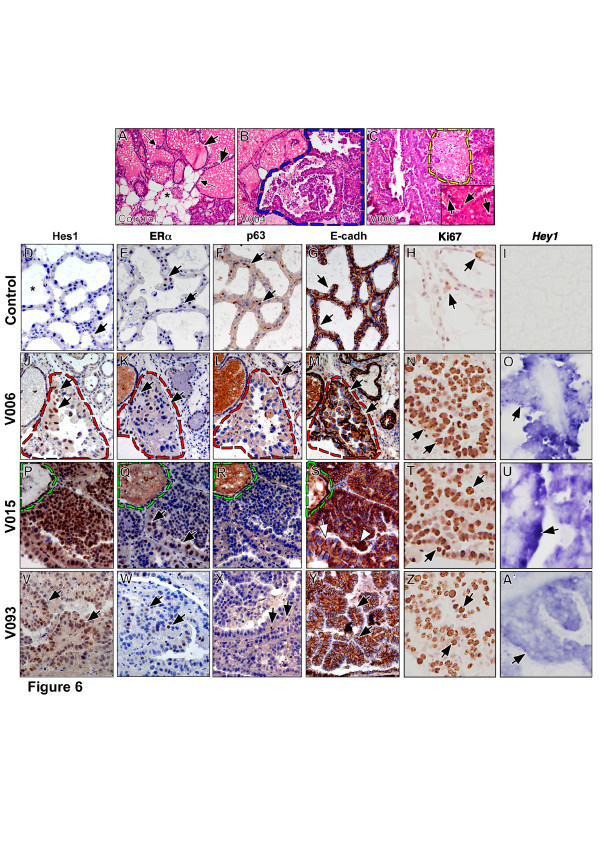
***MMTV-Cre; N1ICD *transgenic mice develop papillary tumors**. (**A-C**) H&E stainings; (**D-H, J-N, P-T, V-Z**) immunostainings; (**I, O, U, A'**) *in situ *hybridization. In (D-G, J-M, P-S,V-Y), sections are counterstained with toluidine blue; in (H, N, T, Z) sections are counterstained with Mayer's Hematoxylin. (A) Normal lactating breast. Greatly expanded secretory lobules (arrows) containing milky secretion are surrounded by breast epithelium (small arrows). Adipose tissue containing lipid vesicles (asterisk) is also abundant. (B) Transgenic female V004. Tumor tissue coexisting with normal tissue is demarcated by a blue dashed line. Breast architecture is widely disorganized. (C) Transgenic female V006. Large necrotic areas (yellow dash line) are observed. Inset shows mitosis (arrows) in tumor tissue. (D) Hes1 expression (brown nuclei, arrow) is rare in normal breast epithelium. Asterisk indicates milky secretion. (E, F) estrogen receptor (brown nuclei, arrows in E) and p63 expression (brown nuclei, arrows in F) in normal breast epithelium. (G) E-cadherin expression in the membrane of breast epithelial cells. (H) Few signs of proliferation in normal lactating breast as indicated by Ki67 staining in only a few cells (brown nuclei, arrows). (I) Normal lactating breasts show no *Hey1 *expression. (J-O) V006 transgenic female. Tumor area is demarcated by a red dashed line. Note the increased Hes1 expression (J, brown nuclei, arrows). (K) estrogen receptor expression (arrows). (L) p63 expression is observed in the non-pathological tissue (arrow) surrounding the tumor. (M) E-cadherin is expressed in membrane of epithelium. (N) Proliferation can be observed throughout the tumor by Ki67 staining (brown nuclei, arrows). (O) Expression of *Hey1 *in tumor tissue (arrow). (P-U) V015 transgenic female. Normal tissue is demarcated by green dashed line. (P) Hes1 expression is strongly up-regulated in tumor cells. (Q) Estrogen receptor (arrows) expression is more widespread. (R) p63 expression is very reduced in tumor tissue. (S) E-cadherin is expressed in membrane (arrow) but also in cytoplasm (arrowheads) of tumor cells. (T) Strong proliferation shown by Ki67 staining (arrows). (U) *Hey1 *expression in tumor (arrows). (V-A') V093 transgenic female. (V) Widespread Hes1 expression in tumor (brown nuclei, arrows). Estrogen receptor (W, arrows) and p63 expression (X, arrows) in tumor. (Y) E-cadherin expression in tumor (arrows). (Z) Strong proliferation in tumor shown by Ki67 staining (arrows). (A') Expression of *Hey1 *in tumor (arrow).

To test *in vivo *the effect of NOTCH activation in the mammary gland we bred the mouse mammary tumor virus (*MMTV)LTR-Cre *transgenic line (*MMTV-Cre*) [20] with the *Rosa26N1ICD *line that expresses the active form of Notch1 (N1ICD) in a conditional manner [21]. With the *MMTV-Cre *driver we targeted N1ICD expression to the secretory epithelium of the mammary gland of pregnant and lactating females. *MMTV-Cre/+;N1ICD/+ *double transgenic mice developed normally and were born at Mendelian ratios (data not shown). Adult *MMTV-Cre/+; N1ICD/+ *females showed high incidence of papillary breast carcinoma (>90%; Additional file 6, Table S1). Figure 6A shows a wildtype lactating breast, with greatly expanded secretory lobules composed of multiple distended acini. Figure 6B, C shows papillary tumors developed in lactating transgenic females (V004 and V006) after three to four rounds of pregnancy and lactation. Tissue architecture was disorganized and large necrotic areas were observed (Figure 6B). Also, frequent mitotic figures and cytological atypia were common (Figure 6C, inset). We analyzed the expression of the Notch targets Hes1 and Hey1, estrogen receptor, the myoepithelial marker p63, which stains basal/myopithelial preserved cells in normal or non-malignant breast tissue [38], the epithelial marker E-cadherin and the cell proliferation marker Ki67. Hes1 expression was low and restricted to a few cells in normal breast epithelial tissue (Figure 6D), like that of estrogen receptor (Figure 6E), while p63 stained around 30% of cells, as expected for a normal tissue (Figure 6F). E-cadherin was strongly expressed in the membrane of normal breast epithelial cells (Figure 6G). Ki67 was expressed only in a few cells in the normal breast (Figure 6H) and *Hey1 *was undetectable (Figure 6I). In the tumors generated in double transgenic *MMTV-Cre;N1ICD *mice (Additional file 6, Table S1) three patterns could be distinguished. The first one, represented by female V006 showed moderate but widespread Hes1 expression (Figure 6J), moderate estrogen receptor staining (Figure 6K), undetectable p63 expression (Figure 6L), normal E-cadherin distribution (Figure 6M), and a marked up-regulation of Ki67 and *Hey1 *expression (Figure 6N, O) in the tumor area. The second one, exemplified by female V015, showed strong and widely distributed Hes1 expression (Figure 6P), relatively high estrogen receptor expression (Figure 6Q) and undetectable p63 expression (Figure 6R) in the tumor. E-cadherin was found in both membrane and cytoplasm (Figure 6S), while Ki67 and *Hey1 *expression was strong (Figure 6T, U). The third pattern was represented by female V093, in which Hes1 expression was widespread but not as much as in the V015 female (Figure 6V), moderate estrogen receptor expression (Figure 6W), very low or absent p63 staining (Figure 6X), with relatively normal E-cadherin expression (Figure 6Y) and Ki67 and Hey1 expression was moderate (Figure 6Z, A'). In general, our data showed that Hes1 and Hey1, as markers of Notch1 activation in epithelial tissues, coexisted with higher estrogen receptor expression. These results are compatible with those obtained with the xenografts of inducible MCF-7-N1ICD cells, whose growth was dependent on N1ICD and estrogens (Figure 4A, B). This observation, together with the fact that tumors were only observed after three to four pregnancies, suggested that the involvement of Notch in breast tumor formation was strongly dependent on estrogens and that Notch expression could lead to changes in estrogen receptor expression.

## Discussion

There is considerable recent interest in understanding how NOTCH signaling affects the development or progression of breast cancer. Notch is critical in mammary gland development, probably by regulating mammary stem cell function [39]. In addition, NOTCH activity has been associated with a number of pro-tumorogenic activities in breast cancer cell lines, and could cause mammary hyperplasia and carcinogenesis in mice [11,40-42]. This evidence strongly pinpoints NOTCH receptors and/or ligands as targets in breast cancer.

Here we used three different *in vitro *and *in vivo *models to analyze the impact of NOTCH signaling in the onset and progression of breast tumors. We found a positive association of NOTCH activity with cancer growth or initiation. In agreement with others [43-46], one of the most consistent observations along our study was the association between NOTCH1 activity and *E-CADHERIN *down-regulation. First, stable expression of activated Notch (N1ICD) was associated with a reduction and delocalization of E-CADHERIN in most of the MCF-7 cell clones analyzed; second, the data with the inducible MCF-7-N1ICD clone B12 clearly established a causal relationship between active NOTCH1 and reduced E-CADHERIN levels; third, inhibition of endogenous NOTCH activation with DAPT in MDA-MB-231 cells, a highly invasive cell line that expresses high NOTCH1 levels, resulted in an increase of E-CADHERIN expression; and fourth, papillary tumors raised in *MMTV-Cre/+;N1ICD/+ *transgenic mice expressing high levels of Hes1 also showed, at least, a delocalization of E-cadherin in the epithelium. Repression and/or delocalization of E-CADHERIN is usually associated with adherens junctions disassembly [29] and enhanced cell invasiveness [33]. Concurrently, we observed that N1ICD-induced *E-CADHERIN *repression correlates with enhanced motility in transwell assays, whereas inhibition of Notch signaling, either by DAPT or RO inhibitors treatment or *DN-CBF1 *overexpression, reduced the motility of the invasive MDA-MB-231 cells. Collectively, these results indicate that NOTCH1 activation could induce EMT in epithelial tumor cells and, consequently, to favor tumor metastasis [47].

The role of Notch as a critical inducer of EMT has been demonstrated during the formation of the cardiac valve primordium [48]. In this process, Notch activates *Snail *expression that in turn down-regulates *VE-cadherin *[48]. We analyzed *Snail1 *expression in the MCF-7 clones expressing N1ICD, and found no clear correlation among N1ICD expression, *E-CADHERIN *down-regulation and *SNAIL1 *expression. We observed a markedly increased *HES1 *and *HEY1 *expression in response to NOTCH1 activation that correlated with a reduction in *E-CADHERIN *expression in both our cellular and animal models. Interestingly, both HES1 and HEY1 have been implicated as part of the hypoxic response associated to breast cancer progression [45].

We also analyzed whether NOTCH1 affects the growth and/or the onset of breast tumors. NOTCH signaling regulates the balance between cell proliferation, differentiation and apoptosis [2] and different reports have demonstrated that NOTCH triggers the proliferation of breast cancer cells [46,49]. In line with these observations, we found that the growth of subcutaneous and orthotopic xenografts produced with MCF-7-B12 cells was boosted after the induction of N1ICD (Figure 5). This effect was cell autonomous, since silencing of N1ICD-expressing tumors by administration of DOXY stopped the growth of tumors whereas induction of N1ICD by DOXY withdrawal boosted tumor growth, with kinetics compatible with N1ICD induction.

Although induction of N1ICD in MCF-7 fosters tumor growth, this effect was only observed when mice were treated with estrogens; indeed, MCF-7 xenografts did not grow in the absence of estrogens, independently of the induction of N1ICD. These results suggest that N1ICD cooperates with the estrogen receptor (ER) on tumor growth, as recently reported [50]. In agreement with this conclusion, *MMTV-Cre/+;N1ICD/+ *mice only developed mammary tumors after repeated pregnancies. It is noteworthy to mention that these breast tumors appeared in the lactating gland and regressed after gland involution; the regression was independent of N1ICD activity as determined by the Hes1 expression level. Our results thus resemble those obtained by Kiaris *et al*. [11], and contrast with the formation of non-regressing mammary neoplasm in transgenic mice expressing the active forms of Notch1, -3 and -4, reported by others [12,41,42]. In summary, our results confirm NOTCH1 as an EMT inducer in breast cancer cells, which may have implications in tumor growth, dissemination and metastasis. The identification of specific factors interacting with NOTCH signaling could thus be relevant to fully understand the role of NOTCH in breast neoplasia.

## Conclusions

NOTCH1 activation attenuates *E-CADHERIN *expression and favors the motility and invasive ability of epithelial human breast cancer MCF-7 cells *in vitro*. In xenografts and in transgenic mice, NOTCH1 activation caused tumors whose increased growth is NOTCH- and estrogen receptor-dependent. To the contrary, NOTCH inhibition leads to increased *E-CADHERIN *expression and attenuates the migratory properties of invasive MDA-MB-231 breast cancer cells. Our findings in these mammary tumor models point to NOTCH1 as a potential therapeutic target in breast cancer onset and progression.

## Abbreviations

CBF1: CSL: Suppressor of Hairless; CSL: CBF1: Suppressor of Hairless: Lag-1; DAB: 3,3'-Diaminobenzidine; DAPI: 4,6-Diamidino-2-phenylindol; DAPT: N-(N-(3,5-difluorophenacetyl)-l-alanyl)-S-phenylglycine t-butyl ester; DMSO: dimethyl sulfoxide; DOXY: doxycycline; ECAD: E-CADHERIN; EMT: epithelial-mesenchymal transition; EE2: 17α-ethynyl estradiol; ERα: estrogen receptor alpha; H&E: Hematoxylin and eosin; Hes1: hairy and enhancer of split 1; Hey1: hairy/enhancer-of-split related with YRPW motif one; HT-29: Human colon adenocarcinoma grade II cell line; IGF-1: Insulin growth factor one; MCF-7: Michigan Cancer Foundation-7 breast cancer cell line; MDA-MB-231: Breast cancer cell line derived from metastatic site (pleural effusion); MMTV-Cre: Mouse mammary Tumor Virus-driven Cre recombinase; N1ICD: Notch one intracellular domain; RO4929097: (2,2-dimethyl-N-(S)-6-oxo-6,7-dihydro-5H-dibenzo(b,d)azepin-7-yl)-N'-(2,2,3,3,3-pentafluoro-propyl)-malonamide); SEM: standard error of the mean; SMC3: Structural Maintenance of Chromomosomes-3 protein; SNA1: SNAIL1; SDF1α: Stromal cell Derived factor 1α; Tet-Off: tetracycline-Off system

## Competing interests

The authors declare that they have no competing interests.

## Authors' contributions

VB, EM, BMP, GL and MC carried out all the experiments. VB, EM, BMP, MC, CMA, SM and JLdlP designed the experiments and analyzed the data. SM and JLdlP wrote the manuscript. All authors read and approved the manuscript.

## Supplementary Material

Additional file 1 **Supplementary Materials and Methods**. Click here for file

Additional file 2 **Figure S1**. *NOTCH4 *expression in the stable MCF-7 clones E8 and F7 compared to the mock MCF-7 cells, measured by qPCR. cDNA of HUVEC cells was used as positive control of *NOTCH4 *expressing cells. Data are mean ± SEM of triplicates in two independents experiments (***P<0.001 determined by student's *t *test). Click here for file

Additional file 3 **Figure S2**. (A) Western Blot showing the expression of N1ICD and E-cadherin in the stable HT-29 clones E11, E12, G12 and G9 compared with mock HT-29 cells; b-actin was used as loading control. (B) Western Blot was quantified by densitometry and the ratio of E-CADHERIN/β-ACTIN was calculated and referred to the mock cells. Click here for file

Additional file 4 **Figure S3**. The inducible clone MCF-7-B12 shows increased VIMENTIN expression while *TWIST1 *is unaffected upon N1ICD induction. (A) Western blot showing inducible N1ICD expression in MCF-7-B12 cells cultured in the presence (+Doxy) or absence (-Doxy) of doxycycline. VIMENTIN expression is increased. (B) Semiquantitative RT-PCR showing that *TWIST1 *transcription does not change after 7 days of N1ICD induction. Click here for file

Additional file 5 **Figure S4**. E-CADHERIN analysis of MCF-7 and MDA-MB-231 cells by flow cytometry. (A) E-CADHERIN staining of MCF-7 cells (black line) and MDA-MB-231 cells (dotted line) is shown. Ten-fold reduction of the mean fluorescence intensity (MnIX) is shown. Gray graph correspond to the staining of both cell lines with the corresponding IgG1 isotype control. (B) Raw data of the MCF-7 analysis (right) compared to the negative control (left). (C) Raw data of the MDA-MB-231 analysis (right) compared to the negative control. Note that there is a ten-fold reduction both in the staining intensity but also in the number of MDA-MB-231 cells staining for E-CADHERIN (79.6%) in comparison with MCF-7 (99.4%). Click here for file

Additional file 6 **Table S1**. Breast tumor formation in *MMTV-Cre/+; N1ICD/+ *females. Click here for file

## References

[B1] Artavanis-Tsakonas S Rand MD Lake RJ Notch signaling: cell fate control and signal integration in development Science 1999 15 770 776 10.1126/science.284.5415.770 10221902

[B2] Bolos V Grego-Bessa J de la Pompa JL Notch signaling in development and cancer Endocr Rev 2007 15 339 363 10.1210/er.2006-0046 17409286

[B3] Harrison H Farnie G Brennan KR Clarke RB Breast cancer stem cells: something out of notching? Cancer Res 2010 15 8973 8976 10.1158/0008-5472.CAN-10-1559 21045140

[B4] Ellisen LW Bird J West DC Soreng AL Reynolds TC Smith SD Sklar J TAN-1, the human homolog of the Drosophila notch gene, is broken by chromosomal translocations in T lymphoblastic neoplasms Cell 1991 15 649 661 10.1016/0092-8674(91)90111-B 1831692

[B5] Robinson DR Kalyana-Sundaram S Wu YM Shankar S Cao X Ateeq B Asangani IA Iyer M Maher CA Grasso CS Lonigro RJ Quist M Siddiqui J Mehra R Jing X Giordano TJ Sabel MS Kleer CG Palanisamy N Natrajan R Lambros MB Reis-Filho JS Kumar-Sinha C Chinnaiyan AM Functionally recurrent rearrangements of the MAST kinase and Notch gene families in breast cancer Nat Med 2011 15 1646 1651 10.1038/nm.2580 22101766 PMC3233654

[B6] Stylianou S Clarke RB Brennan K Aberrant activation of notch signaling in human breast cancer Cancer Res 2006 15 1517 1525 10.1158/0008-5472.CAN-05-3054 16452208

[B7] Ronchini C Capobianco AJ Notch(ic)-ER chimeras display hormone-dependent transformation, nuclear accumulation, phosphorylation and CBF1 activation Oncogene 2000 15 3914 3924 10.1038/sj.onc.1203719 10951584

[B8] Reedijk M Odorcic S Chang L Zhang H Miller N McCready DR Lockwood G Egan SE High-level coexpression of JAG1 and NOTCH1 is observed in human breast cancer and is associated with poor overall survival Cancer Res 2005 15 8530 8537 10.1158/0008-5472.CAN-05-1069 16166334

[B9] Devgan V Mammucari C Millar SE Brisken C Dotto GP p21WAF1/Cip1 is a negative transcriptional regulator of Wnt4 expression downstream of Notch1 activation Genes Dev 2005 15 1485 1495 10.1101/gad.341405 15964998 PMC1151665

[B10] Dievart A Beaulieu N Jolicoeur P Involvement of Notch1 in the development of mouse mammary tumors Oncogene 1999 15 5973 5981 10.1038/sj.onc.1202991 10557086

[B11] Kiaris H Politi K Grimm LM Szabolcs M Fisher P Efstratiadis A Artavanis-Tsakonas S Modulation of notch signaling elicits signature tumors and inhibits hras1-induced oncogenesis in the mouse mammary epithelium Am J Pathol 2004 15 695 705 10.1016/S0002-9440(10)63333-0 15277242 PMC1618582

[B12] Hu C Dievart A Lupien M Calvo E Tremblay G Jolicoeur P Overexpression of activated murine Notch1 and Notch3 in transgenic mice blocks mammary gland development and induces mammary tumors Am J Pathol 2006 15 973 990 10.2353/ajpath.2006.050416 16507912 PMC1606519

[B13] Klinakis A Szabolcs M Politi K Kiaris H Artavanis-Tsakonas S Efstratiadis A Myc is a Notch1 transcriptional target and a requisite for Notch1-induced mammary tumorigenesis in mice Proc Natl Acad Sci USA 2006 15 9262 9267 10.1073/pnas.0603371103 16751266 PMC1570422

[B14] Weijzen S Rizzo P Braid M Vaishnav R Jonkheer SM Zlobin A Osborne BA Gottipati S Aster JC Hahn WC Rudolf M Siziopikou K Kast WM Miele L Activation of Notch-1 signaling maintains the neoplastic phenotype in human Ras-transformed cells Nat Med 2002 15 979 986 10.1038/nm754 12185362

[B15] Milner LA Bigas A Kopan R Brashem-Stein C Bernstein ID Martin DI Inhibition of granulocytic differentiation by mNotch1 Proc Natl Acad Sci USA 1996 15 13014 13019 10.1073/pnas.93.23.13014 8917536 PMC24038

[B16] Chen Q Chen TJ Letourneau PC Costa Lda F Schubert D Modifier of cell adhesion regulates N-cadherin-mediated cell-cell adhesion and neurite outgrowth J Neurosci 2005 15 281 290 10.1523/JNEUROSCI.3692-04.2005 15647471 PMC6725471

[B17] Jarriault S Brou C Logeat F Schroeter EH Kopan R Israel A Signalling downstream of activated mammalian Notch [see comments] Nature 1995 15 355 358 10.1038/377355a0 7566092

[B18] McKenzie GJ Stevenson P Ward G Papadia S Bading H Chawla S Privalsky M Hardingham GE Nuclear Ca2+ and CaM kinase IV specify hormonal- and Notch-responsiveness J Neurochem 2005 15 171 185 10.1111/j.1471-4159.2005.03010.x 15773917

[B19] Kanzler B Kuschert SJ Liu YH Mallo M Hoxa-2 restricts the chondrogenic domain and inhibits bone formation during development of the branchial area Development 1998 15 2587 2597 9636074 10.1242/dev.125.14.2587

[B20] Wagner KU McAllister K Ward T Davis B Wiseman R Hennighausen L Spatial and temporal expression of the Cre gene under the control of the MMTV-LTR in different lines of transgenic mice Transgenic Res 2001 15 545 553 10.1023/A:1013063514007 11817542

[B21] Murtaugh LC Stanger BZ Kwan KM Melton DA Notch signaling controls multiple steps of pancreatic differentiation Proc Natl Acad Sci USA 2003 15 14920 14925 10.1073/pnas.2436557100 14657333 PMC299853

[B22] Soule HD Vazguez J Long A Albert S Brennan M A human cell line from a pleural effusion derived from a breast carcinoma J Natl Cancer Inst 1973 15 1409 1416 4357757 10.1093/jnci/51.5.1409

[B23] Reedijk M Pinnaduwage D Dickson BC Mulligan AM Zhang H Bull SB O'Malley FP Egan SE Andrulis IL JAG1 expression is associated with a basal phenotype and recurrence in lymph node-negative breast cancer Breast Cancer Res Treat 2008 15 439 448 10.1007/s10549-007-9805-3 17990101

[B24] Callahan R Raafat A Notch signaling in mammary gland tumorigenesis J Mammary Gland Biol Neoplasia 2001 15 23 36 10.1023/A:1009512414430 11467450

[B25] Speiser J Foreman K Drinka E Godellas C Perez C Salhadar A Ersahin C Rajan P Notch-1 and Notch-4 biomarker expression in triple-negative breast cancer Int J Surg Pathol 2012 15 139 145 22084425 10.1177/1066896911427035

[B26] Clementz AG Rogowski A Pandya K Miele L Osipo C NOTCH-1 and NOTCH-4 are novel gene targets of PEA3 in breast cancer: novel therapeutic implications Breast Cancer Res 2011 15 R63 10.1186/bcr2900 21679465 PMC3218952

[B27] Ma L Lu MF Schwartz RJ Martin JF Bmp2 is essential for cardiac cushion epithelial-mesenchymal transition and myocardial patterning Development 2005 15 5601 5611 10.1242/dev.02156 16314491

[B28] Luna-Zurita L Prados B Grego-Bessa J Luxan G del Monte G Benguria A Adams RH Perez-Pomares JM de la Pompa JL Integration of a Notch-dependent mesenchymal gene program and Bmp2-driven cell invasiveness regulates murine cardiac valve formation J Clin Invest 2010 15 3493 3507 10.1172/JCI42666 20890042 PMC2947227

[B29] Marambaud P Shioi J Serban G Georgakopoulos A Sarner S Nagy V Baki L Wen P Efthimiopoulos S Shao Z Wisniewski T Robakis NK A presenilin-1/gamma-secretase cleavage releases the E-cadherin intracellular domain and regulates disassembly of adherens junctions EMBO J 2002 15 1948 1956 10.1093/emboj/21.8.1948 11953314 PMC125968

[B30] Mira E Lacalle RA Gonzalez MA Gomez-Mouton C Abad JL Bernad A Martinez AC Manes S A role for chemokine receptor transactivation in growth factor signaling EMBO Rep 2001 15 151 156 10.1093/embo-reports/kve027 11258708 PMC1083823

[B31] Chu D Wang W Xie H Li Y Dong G Xu C Chen D Zheng J Li M Lu Z Ji G Notch1 expression in colorectal carcinoma determines tumor differentiation status J Gastrointest Surg 2009 15 253 260 10.1007/s11605-008-0689-2 18777195

[B32] Noah TK Shroyer NF Notch in the intestine: regulation of homeostasis and pathogenesis Annu Rev Physiol 2013 15 263 288 10.1146/annurev-physiol-030212-183741 23190077

[B33] Peinado H Portillo F Cano A Transcriptional regulation of cadherins during development and carcinogenesis Int J Dev Biol 2004 15 365 375 10.1387/ijdb.041794hp 15349812

[B34] Cailleau R Young R Olive M Reeves WJ Jr Breast tumor cell lines from pleural effusions J Natl Cancer Inst 1974 15 661 674 4412247 10.1093/jnci/53.3.661 PMC7364228

[B35] Dovey HF John V Anderson JP Chen LZ de Saint Andrieu P Fang LY Freedman SB Folmer B Goldbach E Holsztynska EJ Hu KL Johnson-Wood KL Kennedy SL Kholodenko D Knops JE Latimer LH Lee M Liao Z Lieberburg IM Motter RN Mutter LC Nietz J Quinn KP Sacchi KL Seubert PA Shopp GM Thorsett ED Tung JS Wu J Yang S Functional gamma-secretase inhibitors reduce beta-amyloid peptide levels in brain J Neurochem 2001 15 173 181 11145990 10.1046/j.1471-4159.2001.00012.x

[B36] Huynh C Poliseno L Segura MF Medicherla R Haimovic A Menendez S Shang S Pavlick A Shao Y Darvishian F Boylan JF Osman I Hernando E The novel gamma secretase inhibitor RO4929097 reduces the tumor initiating potential of melanoma PLoS One 2011 15 e25264 10.1371/journal.pone.0025264 21980408 PMC3182998

[B37] Manes S Mira E Colomer R Montero S Real LM Gomez-Mouton C Jimenez-Baranda S Garzon A Lacalle RA Harshman K Ruíz A Martínez-A C CCR5 expression influences the progression of human breast cancer in a p53-dependent manner J Exp Med 2003 15 1381 1389 10.1084/jem.20030580 14597737 PMC2194244

[B38] Ribeiro-Silva A Ramalho LN Garcia SB Zucoloto S Does the correlation between EBNA-1 and p63 expression in breast carcinomas provide a clue to tumorigenesis in Epstein-Barr virus-related breast malignancies? Braz J Med Biol Res 2004 15 89 95 14689049 10.1590/s0100-879x2004000100013

[B39] Bouras T Pal B Vaillant F Harburg G Asselin-Labat ML Oakes SR Lindeman GJ Visvader JE Notch signaling regulates mammary stem cell function and luminal cell-fate commitment Cell Stem Cell 2008 15 429 441 10.1016/j.stem.2008.08.001 18940734

[B40] Callahan R Egan SE Notch signaling in mammary development and oncogenesis J Mammary Gland Biol Neoplasia 2004 15 145 163 15300010 10.1023/B:JOMG.0000037159.63644.81

[B41] Jhappan C Gallahan D Stahle C Chu E Smith GH Merlino G Callahan R Expression of an activated Notch-related int-3 transgene interferes with cell differentiation and induces neoplastic transformation in mammary and salivary glands Genes Dev 1992 15 345 355 10.1101/gad.6.3.345 1372276

[B42] Gallahan D Jhappan C Robinson G Hennighausen L Sharp R Kordon E Callahan R Merlino G Smith GH Expression of a truncated Int3 gene in developing secretory mammary epithelium specifically retards lobular differentiation resulting in tumorigenesis Cancer Res 1996 15 1775 1785 8620493

[B43] Leong KG Niessen K Kulic I Raouf A Eaves C Pollet I Karsan A Jagged1-mediated Notch activation induces epithelial-to-mesenchymal transition through Slug-induced repression of E-cadherin J Exp Med 2007 15 2935 2948 10.1084/jem.20071082 17984306 PMC2118507

[B44] Saad S Stanners SR Yong R Tang O Pollock CA Notch mediated epithelial to mesenchymal transformation is associated with increased expression of the Snail transcription factor Int J Biochem Cell Biol 2010 15 1115 1122 10.1016/j.biocel.2010.03.016 20348013

[B45] Chen J Imanaka N Griffin JD Hypoxia potentiates Notch signaling in breast cancer leading to decreased E-cadherin expression and increased cell migration and invasion Br J Cancer 2010 15 351 360 10.1038/sj.bjc.6605486 20010940 PMC2816657

[B46] Rizzo P Miao H D'Souza G Osipo C Song LL Yun J Zhao H Mascarenhas J Wyatt D Antico G Hao L Yao K Rajan P Hicks C Siziopikou K Selvaggi S Bashir A Bhandari D Marchese A Lendahl U Qin JZ Tonetti DA Albain K Nickoloff BJ Miele L Cross-talk between notch and the estrogen receptor in breast cancer suggests novel therapeutic approaches Cancer Res 2008 15 5226 5235 Erratum in Cancer Res 2008, 68:7246. Song, Lynda L [added] 10.1158/0008-5472.CAN-07-5744 18593923 PMC4445363

[B47] Vernon AE LaBonne C Tumor metastasis: a new twist on epithelial-mesenchymal transitions Curr Biol 2004 15 R719 721 10.1016/j.cub.2004.08.048 15341765

[B48] Timmerman LA Grego-Bessa J Raya A Bertrán E Pérez-Pomares JM Díez J Aranda S Palomo S McCormick F Izpisúa-Belmonte JC de la Pompa JL Notch promotes epithelial-mesenchymal transition during cardiac development and oncogenic transformation Genes Dev 2004 15 99 115 10.1101/gad.276304 14701881 PMC314285

[B49] Yamaguchi N Oyama T Ito E Satoh H Azuma S Hayashi M Shimizu K Honma R Yanagisawa Y Nishikawa A Kawamura M Imai J Ohwada S Tatsuta K Inoue J Semba K Watanabe S NOTCH3 signaling pathway plays crucial roles in the proliferation of ErbB2-negative human breast cancer cells Cancer Res 2008 15 1881 1888 10.1158/0008-5472.CAN-07-1597 18339869

[B50] Hao L Rizzo P Osipo C Pannuti A Wyatt D Cheung LW Sonenshein G Osborne BA Miele L Notch-1 activates estrogen receptor-alpha-dependent transcription via IKKalpha in breast cancer cells Oncogene 2010 15 201 213 10.1038/onc.2009.323 19838210 PMC4976641

