# Supplementary material for: Notch activation stimulates migration of breast cancer cells and promotes tumor growth
Source: Breast Cancer Res. 2013 Jul 4;15(4):R54. doi: 10.1186/bcr3447 (PMC3978930; doi:10.1186/bcr3447)
Supplement: Additional file 1 — Supplementary Materials and Methods. [file bcr3447-S1.DOC]

**Additional file 1 - Supplementary Materials and Methods**

**Cell lines and culture conditions.** MCF-7 cells were cultured in MEM (Gibco BRL) with NEAA, sodium pyruvate and L-glutamine. MCF-7 Tet-Off (BD Biosciences) cells were cultured in DMEM with glutamax, 4500 mg/ml glucose and sodium pyruvate (Gibco BRL). MDA-MB-231 cells were cultured in DMEM:Ham´s F12 (1:1) (Gibco BRL) with sodium pyruvate and L-glutamine. HT-29 cells were cultured in DMEM. Media were supplemented with fetal calf serum 10% penicillin and streptomycin. Cells were cultured at 37ºC in a humidified atmosphere and 5% of CO_2_.

**Transfection of MCF-7 and HT-29 cells.** The cDNA fragment encoding the active version of mouse Notch1 (N1ICDΔ^OP^) was used . Stable transfectants of MCF-7 and HT-29 cells were obtained by transfection of pcDNA3-N1ICD or the empty vector with Lipofectamine Plus (Invitrogen). The pcDNA3-N1ICD includes a myc tag fused to the amino-terminus of N1ICD to facilitate its detection. Transfected cells were selected with 1-2 mg/ml of geneticin (Calbiochem) for 3-4 weeks. Three clones overexpressing N1ICD: E8, F5 and F7 were selected for further studies and a pool of cells transfected with the empty vector and selected with geneticin were employed as mock cells. In the case of HT-29 cells, four clones overexpressing N1ICD: E11, E12, G12 and G9 were selected. The Tet-Off system was employed to obtain transfectants of MCF-7 with inducible N1ICD expression. The cell line MCF-7 Tet-Off (BD Biosciences) was transfected with pTRE2purN1ICD and selection with geneticin 100 µg/ml and puromycin (BD Biosciences Clontech) was applied for 3-4 weeks. Doxycycline (SIGMA-Aldrich, 1 µg/ml) was also included in the culture medium to keep off the expression of N1ICD. Three clones were selected for further analysis B12, M5 and M20.

**Western blot analysis.** Cell pellets were used to prepare both total protein extracts with RIPA buffer and cytosolic and nuclear fractions as described in the presence of protease and phosphatases inhibitors. 15-30 µg of protein samples were resolved in PAGE-SDS gels and after transfer to Immobilon-P (Millipore), the filters were incubated with the appropriate antibodies. The antibodies used were: anti-cleaved Notch1 (Cell Signaling), anti-c-Myc (9E10, Sigma), anti-human E-CADHERIN (Calbiochem), anti-HES1 (Chemicon), anti-ERα 1D5 (Dako), anti-β-ACTIN (Sigma), anti-α-TUBULIN (Sigma) and anti-SMC3 (Chemicon). After incubation with the appropriate HRP goat polyclonal antibodies (DAKO Cytomation), ECL or ECL Plus (Amersham) was used for signal detection.

**Immunofluorescence.** MCF-7 cells were grown to confluence on 12 mm diameter coverslips in p60 plates and fixed with 4% PFA for 10 min at RT or with methanol for 30 s at -20ºC. Cells were incubated with the primary antibodies (anti E-CADHERIN (Sigma, clon DECMA-1) and anti-Myc (9E10)) and with the appropriate conjugated secondary antibodies. Samples were mounted with Vectashield with DAPI (Vector Laboratories, Burlingam, CA) and visualized with a Zeiss Axiophot microscope equipped with epifluorescence.

**Immunohistochemistry.** Specimens were fixed in 10% buffered formalin (Sigma) and embedded in paraffin wax. For histopathological studies, 3 μm-thick sections were stained with hematoxylin and eosin (H&E). Additional immunohistochemical examination of the tissues analyzed was performed using specific antibodies against Hes1 (Santa Cruz), E-Cadherin (BD Transduction), ERα (Santa Cruz) or p63 (NeoMarkers) or Ki67 (Dako). Following incubation with the primary antibodies, positive cells were visualized using 3,3-diaminobenzidine tetrahydrochloride plus (DAB+) as a chromogen.

**Flow cytometry.** MCF-7 and MDA-MB-231 cell suspension obtained after trypsinization were incubated with anti E-cadherin (clone 67A4, Immunotech) or IgG1 isotype control followed by the secondary antibody FITC-conjugated anti-mouse IgG1 (SouthernBiotech, Al, USA). Staining was analyzed in an EPICS XL flow cytometer (Coulter Electronics Hialeah, FL).

**References**

1. Milner LA, Bigas A, Kopan R, Brashem-Stein C, Bernstein ID, Martin DI: **Inhibition of granulocytic differentiation by mNotch1**. *Proc Natl Acad Sci U S A* 1996, **93**(23):13014-13019.

2. Andrews NC, Faller DV: **A rapid micropreparation technique for extraction of DNA-binding proteins from limiting numbers of mammalian cells**. *Nucleic Acids Res* 1991, **19**(9):2499.
