# Supplementary material for: Notch activation stimulates migration of breast cancer cells and promotes tumor growth
Source: Breast Cancer Res. 2013 Jul 4;15(4):R54. doi: 10.1186/bcr3447 (PMC3978930; doi:10.1186/bcr3447)

**Additional file 2 - Figure S1.**

**Figure S1 -** *NOTCH4* expression in the stable MCF-7 clones E8 and F7 compared to the mock MCF-7 cells, measured by qPCR. cDNA of HUVEC cells was used as positive control of *NOTCH4* expressing cells. Data are mean ± SEM of triplicates in two independents experiments (***P<0.001 determined by student's *t* test).


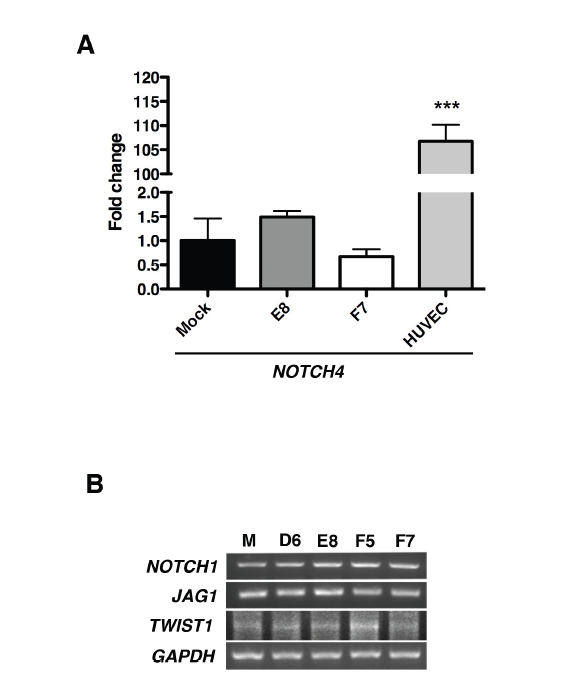

Supplement: Additional file 2 — Figure S1. NOTCH4 expression in the stable MCF-7 clones E8 and F7 compared to the mock MCF-7 cells, measured by qPCR. cDNA of HUVEC cells was used as positive control of NOTCH4 expressing cells. Data are mean ± SEM of triplicates in two independents experiments (***P<0.001 determined by student's t test). [file bcr3447-S2.DOC]
