# Supplementary material for: Notch activation stimulates migration of breast cancer cells and promotes tumor growth
Source: Breast Cancer Res. 2013 Jul 4;15(4):R54. doi: 10.1186/bcr3447 (PMC3978930; doi:10.1186/bcr3447)

**Additional file 3 - Figure S2.**

**Figure S2.** (A) Western Blot showing the expression of N1ICD and E-cadherin in the stable HT-29 clones E11, E12, G12 and G9 compared with mock HT-29 cells; b-actin was used as loading control. (B) Western Blot was quantified by densitometry and the ratio of E-CADHERIN/β-ACTIN was calculated and referred to the mock cells.


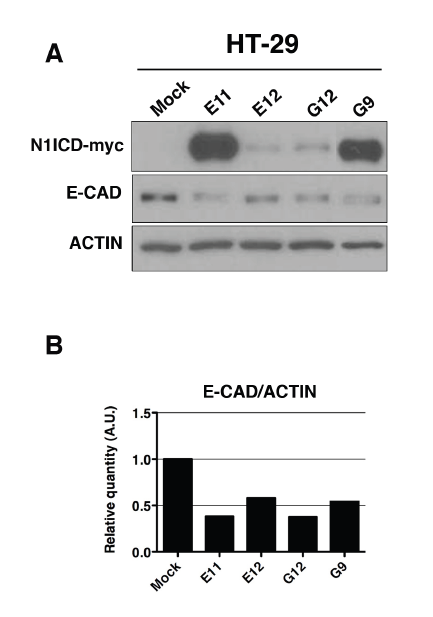

Supplement: Additional file 3 — Figure S2. (A) Western Blot showing the expression of N1ICD and E-cadherin in the stable HT-29 clones E11, E12, G12 and G9 compared with mock HT-29 cells; b-actin was used as loading control. (B) Western Blot was quantified by densitometry and the ratio of E-CADHERIN/β-ACTIN was calculated and referred to the mock cells. [file bcr3447-S3.DOC]
