# Supplementary material for: Notch activation stimulates migration of breast cancer cells and promotes tumor growth
Source: Breast Cancer Res. 2013 Jul 4;15(4):R54. doi: 10.1186/bcr3447 (PMC3978930; doi:10.1186/bcr3447)

**Additional file 4 - Figure S3.**

**Figure S3**. The inducible clone MCF-7-B12 shows increased VIMENTIN expression while *TWIST1* is unaffected upon N1ICD induction. (A) Western blot showing inducible N1ICD expression in MCF-7-B12 cells cultured in the presence (+Doxy) or absence (-Doxy) of doxycycline. VIMENTIN expression is increased. (B) Semiquantitative RT-PCR showing that *TWIST1* transcription does not change after 7 days of N1ICD induction.


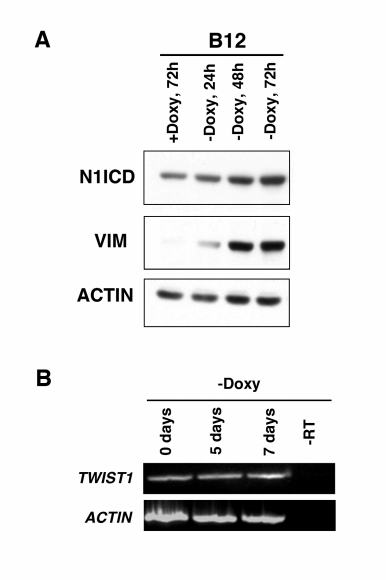

Supplement: Additional file 4 — Figure S3. The inducible clone MCF-7-B12 shows increased VIMENTIN expression while TWIST1 is unaffected upon N1ICD induction. (A) Western blot showing inducible N1ICD expression in MCF-7-B12 cells cultured in the presence (+Doxy) or absence (-Doxy) of doxycycline. VIMENTIN expression is increased. (B) Semiquantitative RT-PCR showing that TWIST1 transcription does not change after 7 days of N1ICD induction. [file bcr3447-S4.DOC]
