# Supplementary material for: Notch activation stimulates migration of breast cancer cells and promotes tumor growth
Source: Breast Cancer Res. 2013 Jul 4;15(4):R54. doi: 10.1186/bcr3447 (PMC3978930; doi:10.1186/bcr3447)

**Additional file 5 - Figure S4.**

**Figure S4**. E-CADHERIN analysis of MCF-7 and MDA-MB-231 cells by flow cytometry. (A) E-CADHERIN staining of MCF-7 cells (black line) and MDA-MB-231 cells (dotted line) is shown. Ten-fold reduction of the mean fluorescence intensity (MnIX) is shown. Gray graph correspond to the staining of both cell lines with the corresponding IgG1 isotype control. (B) Raw data of the MCF-7 analysis (right) compared to the negative control (left). (C) Raw data of the MDA-MB-231 analysis (right) compared to the negative control. Note that there is a ten-fold reduction both in the staining intensity but also in the number of MDA-MB-231 cells staining for E-CADHERIN (79.6%) in comparison with MCF-7 (99.4%).


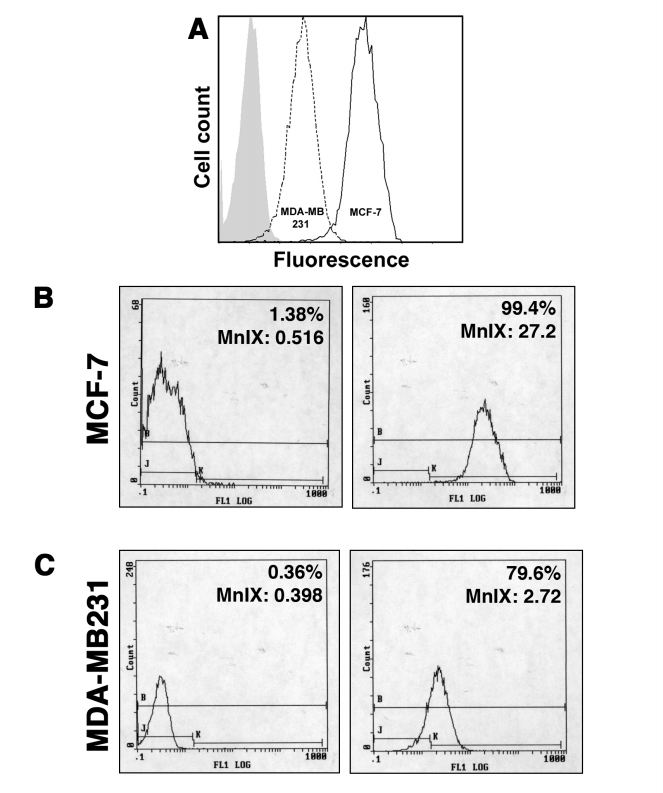

Supplement: Additional file 5 — Figure S4. E-CADHERIN analysis of MCF-7 and MDA-MB-231 cells by flow cytometry. (A) E-CADHERIN staining of MCF-7 cells (black line) and MDA-MB-231 cells (dotted line) is shown. Ten-fold reduction of the mean fluorescence intensity (MnIX) is shown. Gray graph correspond to the staining of both cell lines with the corresponding IgG1 isotype control. (B) Raw data of the MCF-7 analysis (right) compared to the negative control (left). (C) Raw data of the MDA-MB-231 analysis (right) compared to the negative control. Note that there is a ten-fold reduction both in the staining intensity but also in the number of MDA-MB-231 cells staining for E-CADHERIN (79.6%) in comparison with MCF-7 (99.4%). [file bcr3447-S5.DOC]
