# Supplementary material for: Notch activation stimulates migration of breast cancer cells and promotes tumor growth
Source: Breast Cancer Res. 2013 Jul 4;15(4):R54. doi: 10.1186/bcr3447 (PMC3978930; doi:10.1186/bcr3447)
Supplement: Additional file 6 — Table S1. Breast tumor formation in MMTV-Cre/+; N1ICD/+ females. [file bcr3447-S6.DOC]

**Additional file 6 - Table S1. Breast tumor formation in *MMTV-Cre/+; N1ICD/+* females.**

| **Identification** | **Pregnancies** | **Observations** |
| --- | --- | --- |
| V002 | 4 | Tumors in 260 days. |
| V004 | 4 | Tumors in 213 days. |
| V006 | 4 | Tumors in 236 days. |
| V015 | 2 | Tumors in 143 days. |
| V016 | 2 | Tumors in 202 days. |
| V065 | 4 | Tumors in 223 days. |
| V075 | 7 | Tumors in 288 days. |
| V082 | 6 | Apparently normal breasts |
| V093 | 6 | Tumors in 229 days. |
| V149 | 3 | Tumors in 208 days. |
| V167 | 4 | Tumors in 193 days. |
| V121 (Control:  N1ICD/+) | 5 | Apparently normal breasts |
